# Chromomycins A_2_ and A_3_ from Marine Actinomycetes with TRAIL Resistance-Overcoming and Wnt Signal Inhibitory Activities

**DOI:** 10.3390/md12063466

**Published:** 2014-06-05

**Authors:** Kazufumi Toume, Kentaro Tsukahara, Hanako Ito, Midori A. Arai, Masami Ishibashi

**Affiliations:** Department of Natural Products Chemistry, Graduate School of Pharmaceutical Sciences, Chiba University, 1-8-1 Inohana, Chuo-ku, Chiba 260-8675, Japan; E-Mails: toume@faculty.chiba-u.jp (K.T.); kkktt2005@chiba-u.jp (K.T.); z8p1012@students.chiba-u.jp (H.I.); midori_arai@chiba-u.jp (M.A.A.)

**Keywords:** chromomycin A_2_, chromomycin A_3_, actinomycetes, TRAIL, Wnt

## Abstract

A biological screening study of an actinomycetes strain assembly was conducted using a cell-based cytotoxicity assay. The CKK1019 strain was isolated from a sea sand sample. Cytotoxicity-guided fractionation of the CKK1019 strain culture broth, which exhibited cytotoxicity, led to the isolation of chromomycins A_2_ (**1**) and A_3_ (**2**). **1** and **2** showed potent cytotoxicity against the human gastric adenocarcinoma (AGS) cell line (IC_50_ **1**; 1.7 and **2**; 22.1 nM), as well as strong inhibitory effects against TCF/β-catenin transcription (IC_50_ **1**; 1.8 and **2**; 15.9 nM). **2** showed the ability to overcome tumor necrosis factor (TNF)-related apoptosis-inducing ligand (TRAIL) resistance. To the best of our knowledge, the effects of chromomycins A_2_ (**1**) and A_3_ (**2**) on TRAIL resistance-overcoming activity, and on the Wnt signaling pathway, have not been reported previously. Thus, **1** and **2** warrant potential drug lead studies in relation to TRAIL-resistant and Wnt signal-related diseases and offer potentially useful chemical probes for investigating TRAIL resistance and the Wnt signaling pathway.

## 1. Introduction

Natural small molecules from actinomycetes provide a number of antimicrobials and anticancer agents with original and ingenious structures, as well as strong biological activities [1]. As a result, they are widely recognized as a promising resource that can potentially supply new drug discovery lead or seed compounds. We have previously isolated actinomycetes from soil, sea sand and seawater samples collected around Japan in order to investigate the bioactive metabolites obtained from these actinomycetes. Using these assembled actinomycetes, we examined the bioactive metabolites of actinomycetes strains and isolated a rare phenazine [2], its glycosides [3], nonactin derivatives [4], cyclic hydroxamates [5], azaquinone-phenylhydrazone [6] and naphthopyridazone alkaloid [7] from *Streptomyces* sp. Of these compounds, several showed the ability to abrogate tumor necrosis factor (TNF)-related apoptosis-inducing ligand (TRAIL) resistance [6,7,8], along with inhibitory effects on the Wnt signal pathway [4,5].

In our screening program for obtaining bioactive natural products from our actinomycetes assembly, which consists of more than 1200 strains, we detected several strains that showed potent cytotoxicity. Of these strains, we recently examined the active constituents in the fermented broth of the actinomycetes strain, CKK1019, which led to the isolation of chromomycins A_2_ (**1**) and A_3_ (**2**) as bioactive compounds. Chromomycins, members of the aureolic acid family, are known to have antitumor activity and were isolated from soil- and marine-derived actinomycetes [9]. Herein, we will describe the activity-guided isolation and identification of those active compounds.

## 2. Results and Discussion

### 2.1. Isolation and Identification of 1 and 2

Using a cell-based cytotoxicity assay system, we examined the extracts of actinomycetes isolated and cultivated in our laboratory and detected the cytotoxic effects against the human gastric adenocarcinoma (AGS) cell line (34% viability of the control at 10 μg/mL) of the MeOH extract of the CKK1019 actinomycetes strain, which was isolated from a sea sand sample collected at Okinoshima Island, Tateyama City, Chiba Prefecture, Japan. The culture conditions of this strain were then investigated. The strain was cultivated in 4%, 2% and 0% artificial seawater containing liquid Waksman media. After the extraction of culture broth, these extracts exhibited the same retention factor (Rf) value spots, the same pattern on TLC and similar cytotoxicities.

**Figure 1:**
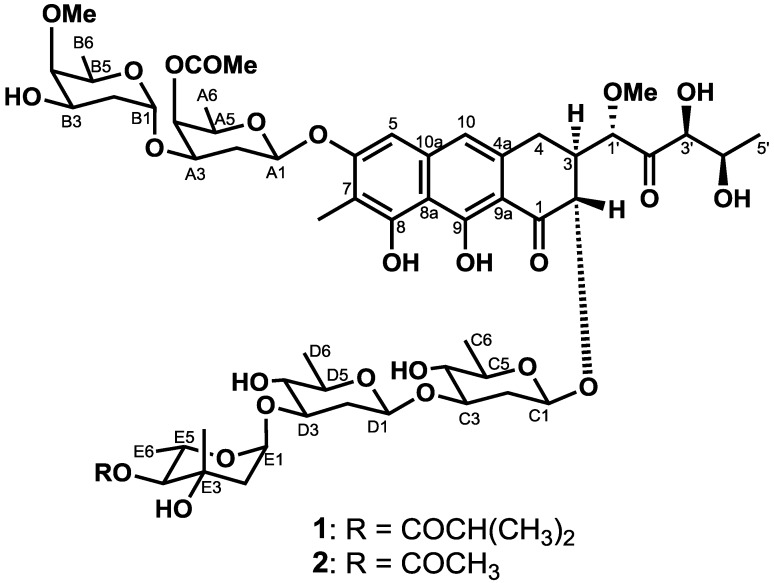
Structures of isolated Compounds **1** and **2**.

Next, large-scale cultivation was conducted using seawater-free liquid Waksman media. EtOAc was used to extract the supernatant of the culture broth (6.2 L). Cytotoxicity-guided fractionation of the EtOAc extract, which showed cytotoxicity, was conducted using silica gel and ODS column chromatography, as well as preparative ODS HPLC. These processes yielded chromomycins A_2_ (**1**, 50 mg) [10,11] and A_3_ (**2**, 86.3 mg) [10] (Figure 1), which were identified on the basis of spectroscopic data, including NMR, MS and optical rotation with references to the literature [10,11].

### 2.2. Biological Activities of 1 and 2

**Figure 2:**
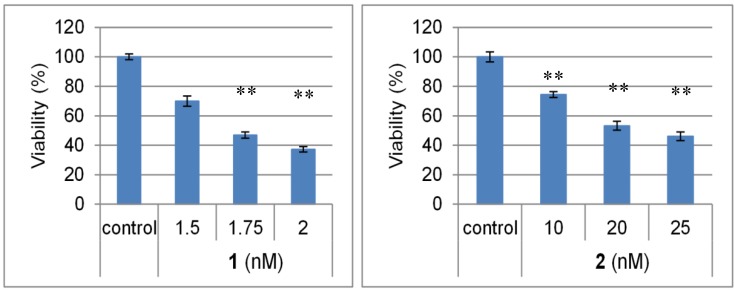
Cytotoxicities of chromomycins A_2_ (**1**) and A_3_ (**2**) against human gastric adenocarcinoma (AGS) cells. AGS cells were treated at the indicated concentration of test samples for 24 h. Cell viability was determined after 24 h by the fluorometric microculture cytotoxicity assay (FMCA). The bars represent the means ± SD (*n* = 3) with significance determined with Tukey’s test. ** *p* < 0.01 *vs.* the control (blank containing 0.1% DMSO without compound).

Chromomycins A_2_ (**1**) and A_3_ (**2**) exhibited potent cytotoxicity against the AGS cell line, with IC_50_ values of 1.7 and 22.1 nM, respectively (Figure 2).

**Figure 3:**
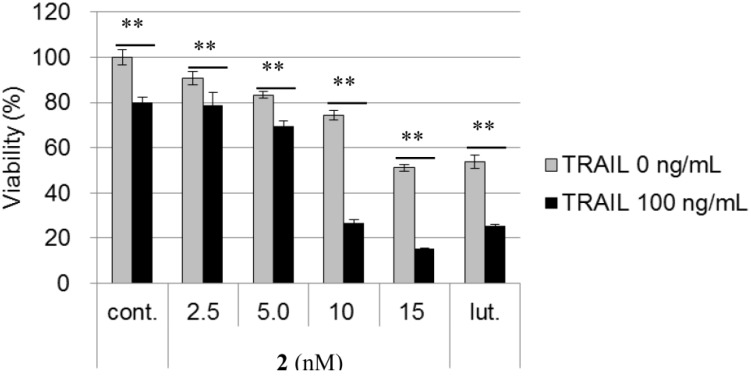
TNF-related apoptosis-inducing ligand (TRAIL) resistance-overcoming activity of chromomycin A_3_ (**2**) in AGS cells. AGS cells were treated at the indicated concentration of test samples and/or 100 ng/mL of TRAIL for 24 h. Cell viability was determined after 24 h by a FMCA assay. The bars represent means ± SD (*n* = 3) with significance determined with Tukey’s test. ** *p* < 0.01 *vs.* the control (blank containing 0.1% DMSO without compound).

The AGS cell line is known to be resistant against the tumor necrosis factor (TNF)-related apoptosis-inducing ligand (TRAIL) [12]. TRAIL, a member of the TNF superfamily, is considered to be a promising anti-cancer agent, due to its ability to cause tumor selective apoptosis. TRAIL binds to death receptors, such as death receptor 5 (DR5) and/or death receptor 4 (DR4), which allows the formation of a death-inducing signaling complex (DISC), resulting in the activation of caspase-signaling pathways and, ultimately, apoptosis [13]. However, large numbers of cancer cells, especially highly malignant tumors, are resistant to TRAIL. Therefore, identifying compounds that can overcome TRAIL-resistance has become an important strategy in the development of new anticancer drugs. The ability of chromomycin A_3_ (**2**) to overcome TRAIL resistance was evaluated using the previously described method [14,15]. As shown in Figure 3, the treatment of AGS cells with 100 ng/mL TRAIL for 24 h resulted in only a slight decrease in cell viability (20%), while luteolin [16] at 17.5 μM, which was used as a positive control, was 35% more potent when administered in combination with TRAIL than when the compound was administrated alone. The treatment of AGS cells with **2** (10 nM) in the presence of TRAIL (100 ng/mL) led to cell viability being 52% lower than that of treatment with the compound alone (without TRAIL), which confirmed the potent abilities of these compounds to overcome TRAIL resistance.

Next, we examined the effects of chromomycins A_2_ (**1**) and A_3_ (**2**) on the Wnt signaling pathway. To the best of our knowledge, the effects of chromomycins on Wnt signaling have not been reported. The Wnt signaling pathway has been highly conserved throughout evolution from metazoans to humans and plays an important role in the regulation of numerous cellular processes, including embryonic development, differentiation, proliferation, survival, polarity, migration and the specification of cell fate in various cells [17]. However, the strong activation of Wnt signaling is a pivotal factor for oncogenesis in various types of cancer [18,19], particularly in human colon cancer. Therefore, Wnt signaling is considered to be an attractive therapeutic target for colon cancer. Furthermore, recent studies revealed that this signaling pathway might play significant roles in supporting differentiation, as well as the formation and maintenance of stem cells [20]. On the other hand, the deregulation of this pathway has also been associated with various human diseases, including diabetes, osteoporosis and Alzheimer’s disease [18]. Therefore, small molecules that have the ability to modulate the Wnt signaling pathway can be useful biological study agents and have the potential to become lead compounds for drug discovery. Using the luciferase reporter gene assay system [21,22], the inhibitory activities of these compounds on TCF/β-catenin transcription were evaluated.

**Figure 4:**
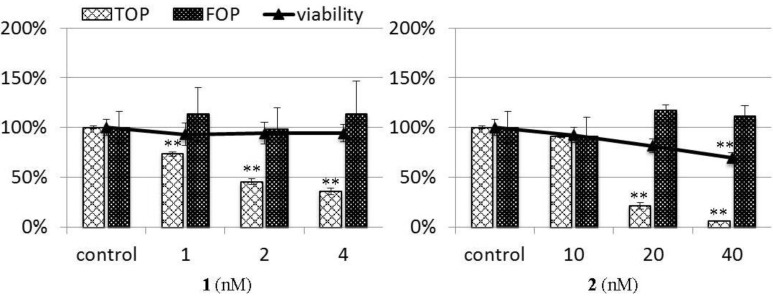
The inhibitory activity of chromomycins A_2_ (**1**) and A_3_ (**2**) on TCF/β-catenin transcription. The TOP-Flash (TOP) and FOP-Flash (FOP) assays were evaluated luciferase assay system using STF (SuperTOP-Flash)/293 and 293 T cells, respectively. Cells were treated with compounds and LiCl (15 mM) for 24 h, then the luciferase activity was measured. The cell viability of STF/293 cells of each treatment (24 h) was also evaluated by the FMCA assay. Data are presented as the mean ± SD. ** *p* < 0.01 *vs.* the control (blank containing 0.1% DMSO without compound).

Wnt signaling activates gene transcription by forming a complex between the DNA-binding proteins of the TCF/LEF family and β-catenin, and that, under this condition, SuperTOP-Flash, a β-catenin-responsive reporter plasmid with seven copies of TCF-binding sites (CCTTTGATC), is activated. STF/293 cells, 293 cells stably expressing the SuperTOP-Flash reporter construct, were used for the TOP-Flash assay. Together with the TOP-Flash assay, the cell viability of STF/293 cells was also tested. Because a decrease in cell number may contribute to the false positive inhibition of TCF/β-catenin transcriptional activity, an inhibitor of TCF/β-catenin transcription may inhibit SuperTOP-Flash activity, but it will not be able to inhibit transcription in SuperFOP-Flash-transfected cells because SuperFOP-Flash has six copies of the mutated TCF-binding sites (CCTTTGGCC). The FOP-Flash assay was conducted using 293 T cells transiently transfected with the SuperFOP-Flash reporter construct. As shown in Figure 4, chromomycins A_2_ (**1**) and A_3_ (**2**) strongly inhibited TCF/β-catenin transcription, with IC_50_ values of 1.8 and 15.9 nM, high viability (>69%) and no significant decrease in SuperFOP-Flash activity. These results indicate that chromomycins A_2_ (**1**) and A_3_ (**2**) are potent TCF/β-catenin transcription inhibitors.

## 3. Experimental Section

### 3.1. General Experimental Procedures

Optical rotations were measured with a JASCO P-1020 polarimeter. NMR spectra were recorded on JEOL ECA600 and ECP400 spectrometers with a deuterated solvent, the chemical shifts of which were used as an internal standard. Electrospray ionization mass spectra (ESIMS) were obtained on a Shimadzu LCMS-2020 spectrometer. Column chromatography was performed using silica gel PSQ100B and Chromatorex ODS (Fuji Silysia Chemical Ltd., Kasugai, Japan). Preparative HPLC was performed using Develosil ODS HG-5 (Nomura Chemical Co., Ltd., Seto, Japan).

### 3.2. Microbial Strain and Fermentation

CKK1019 was separated on humic acid-vitamin (HV) agar [23], a medium for the selective isolation of actinomycetes, from a sea sand sample collected at Okinoshima Island, Tateyama City, Chiba Prefecture, Japan, as described previously [24]. Briefly, the sea sand sample was shaken in water, filtrated using a nitrocellulose membrane filter (pore size: 0.22 μm; GSWP, Merck Millipore Billerica, MA, USA), after which the filter was overlaid on a plate of HV agar. After a three-or four-day incubation, the filter was removed from the agar, and the plate was reincubated until growth was observed. The spores of this strain were kept in a freezer in a 15% glycerol solution, grown on solid Waksman medium containing agar for 3–5 days, transferred to a flask (500 mL) and inoculated into 100 mL of a Waksman medium consisting of glucose (2 g/100 mL), meat extract (0.5 g/100 mL), peptone (0.5 g/100 mL), dried yeast (0.3 g/100 mL), NaCl (0.5 g/100 mL) and CaCO_3_ (0.3 g/100 mL). The mixture was then cultured at 28 °C for three days on a reciprocating shaker. The seed culture (20 mL) was then transferred into a flask (3 L) containing 700 mL of the Waksman medium and cultured at 28 °C for seven days on the reciprocating shaker.

### 3.3. Extraction and Isolation

The culture broth (6.2 L in total) of CKK1019 was harvested and centrifuged (6000 rpm, 15 min) to separate mycelia and the supernatant. The supernatant was concentrated under reduced pressure to approximately 500 mL and partitioned between EtOAc (500 mL × 3) to obtain the EtOAc-soluble fraction (2.4 g). The EtOAc-soluble fractions of the supernatant were subjected to silica gel column chromatography (28 × 250 mm) eluted stepwise (CHCl_3_:MeOH = 100:0, 95:5, 90:10, 85:15, 80:20, 70:30, 60:40, 0:100) to obtain eleven fractions, 1A to 1K. Fractions 1E and 1F eluted with CHCl_3_:MeOH = 95:5 were combined (1043 mg), then subjected to the ODS column (28 × 205 mm), which was eluted with MeOH:H_2_O = 6/4, 65/35, 7/3, 8/2, 1/0 to obtain 13 fractions. Fraction 2G (86.3 mg), which was eluted with MeOH:H_2_O = 7:3, was identified as chromomycin A_3_ (**2**). A portion (67 mg) of Fraction 2J (231 mg), eluted with MeOH:H_2_O = 7:3, was further separated by preparative HPLC (CAPCELL PACK C18 MG-2, 10 × 250 mm; eluent, 55% MeCN in H_2_O; flow rate, 2.0 mL/min; UV detection at 254 nm and Refractive Index (RI) detection) to obtain chromomycin A_2_ (**1**, 50 mg, *t*_R_ 31 min).

Chromomycin A_2_ (**1**): 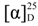 −12 (*c* 1.2 EtOH); ^1^H and ^13^C NMR data, see Table 1; ESIMS *m*/*z* 1233 [M + Na]^+^.

Chromomycin A_3_ (**2**): 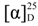 −42 (*c* 1.0 EtOH); ^1^H and ^13^C NMR data, see Table 1; ESIMS *m*/*z* 1205 [M + Na]^+^.

**Table 1:** ^1^H and ^13^C NMR spectroscopic data for chromomycins A_2_ (**1**) and A_3_ (**2**).

Position	1			2	
	δ_H_ (*J* in Hz) ^a^	δ_H_ (*J* in Hz) ^b^	δ_C_^c^	δ_H_ (*J* in Hz) ^b^	δ_C_^c^
1			202.1		202.1
2	4.64, d (11.2)	4.71, d (11.4)	75.9	4.71, d (11.6)	75.9
3	2.58, m	2.58, m	43.7	2.67, m	43.7
4	3.00, d (15.2)	3.06, d (13.1)	26.9	3.13, m	26.9
	2.58, m	2.58, m		2.67, m	
5	6.58, s	6.54, s	100.8	6.60, s	100.7
6			159.6		159.6
7			111.6		111.6
8			156.1		156.1
9			165.3		165.2
10	6.70, s	6.66, s	117.0	6.72, s	117.0
4a			134.6		134.5
8a			108.1		108
9a			108.1		108
10a			138.4		138.3
7-CH_3_	2.10, s	2.16, s	8.2	2.16, s	8.2
8-OH		9.74		9.78, s	
9-OH		15.67		15.68, s	
1′	4.67, s	4.69, br s	81.8	4.70, d (1.6)	81.9
2′			211.2		211.2
3′	4.11, d (1.5)	4.20, br s	78.2	4.21, d (2.0)	78.2
4′	4.21, qd (6.4, 1.5)	4.36, q (6.0)	67.9	4.37, qd (6.4, 2.0)	67.8
5′	1.25 d (6.4)	1.35, d (6.0)	20.5	1.37, d (6.4)	20.5
1′-OCH_3_	3.40, s	3.49, s	59.6	3.50, s	59.6
Sugar A					
A1	5.18, d (8.4)	5.19, d (10.0, 2.0)	97.3	5.21, dd (9.8, 2.1)	97.3
A2	2.16, m	2.20, m	32.9	2.26, m	32.9
	2.00, m	2.02, m		2.26, m	
A3	3.96, m	3.93, m	69.9	4.00, m	69.8
A4	5.10, d (1.7)	5.15, d (2.6)	67.2	5.16, d (3.0)	67.2
A5	3.76, m	3.80, q (6.4)	69.7	3.82, q (6.4)	69.7
A6	1.19, d (6.0)	1.27, d (6.4)	16.8	1.28, d (6.4)	16.8
CH_3_-CO	2.09, s	2.15, s	20.8	2.15, s	20.8
CH_3_-CO			170.9		170.9
Sugar B					
B1	5.05, d (2.8)	5.10, br s	95.2	5.10, d (2.4)	95.1
B2	1.78, m	1.63–1.74, m	33.5	1.63–1.75, m	33.4
	1.64, m	1.63–1.74, m		1.63–1.75, m	
B3	3.89, dd (7.8, 2.8)	3.93, m	65.9	3.93, m	65.8
B4	3.14, d (2.8)	3.21, d (3.2)	81.5	3.21, d (3.2)	81.5
B5	3.82, q (6.5)	3.93, m	66.7	3.89, q (6.4)	66.7
B6	1.14, d (6.5)	1.27, d (6.5)	17.2	1.29, d (6.4)	17.2
B4-OCH_3_	3.51, s	3.57, s	62.3	3.58, s	62.3
Sugar C					
C1	5.01, d (9.2)	5.07, br d (9.7)	100.3	5.08, dd (9.7, 1.6)	100.3
C2	2.46, dd (12.2, 4.6)	2.49, dd (11.4, 5.2)	37.4	2.49, ddd (12.8, 5.1, 1.6)	37.4
	1.64, m	1.63, m		1.75, m	
C3	3.59, m	3.57, m	82.3	3.59, m	82.3
C4	3.06, d (8.8)	3.13, d (9.0)	75.1	3.08, m	75.1
C5	3.27, m	3.38, dq (9.0, 5.5)	72.1	3.32, dq (9.1, 5.5)	72.1
C6	1.28, d (5.6)	1.34, d (5.5)	18.0	1.34, d (5.5)	18.0
Sugar D					
D1	4.55, d (9.6)	4.59, d (9.0)	99.7	4.60, d (9.6, 2.0)	99.7
D2	2.22, dd (11.9, 4.2)	2.27, dd (13.1, 4.8)	37.1	2.29, dd (5.9, 2.0)	37.0
	1.56, d (11.9)	1.63, m		1.75, m	
D3	3.49, m	3.49, m	80.7	3.47, m	80.5
D4	3.03, d (8.0)	3.10, d (8.5)	75.2	3.08, m	75.2
D5	3.27, m	3.30, qd (9.0, 6.0)	72.3	3.40, dq (9.1, 6.0)	72.2
D6	1.31, d (6.0)	1.37, d (6.0)	17.8	1.38, d (6.0)	17.8
Sugar E					
E1	4.96 (t, 2.8)	4.99, dd (3.9, 2.0)	97.1	5.01, dd (4.0, 2.2)	97.0
E2	1.92, m	2.00, m	43.8	2.00, dd (13.8, 4.0)	43.6
E3			70.6	2.06, dd (13.8, 2.2)	70.6
E4	4.55, d (9.6)	4.59, d (9.0)	79.4	4.61, d (9.3)	79.5
E5	3.98, m	3.88, q (6.7)	67.0	4.00, m	67.0
E6	1.22, d (5.2)	1.21, d (7.4)	17.8	1.23, d (6.4)	17.8
E3-CH_3_	1.28, s	1.33, s	22.9		
(CH_3_)_2_CH-CO	1.13, d (6.8)	1.19, d (7.4)	19.0		
(CH_3_)_2_CH-CO	2.58, m	2.56, m	34.2		
(CH_3_)_2_CH-CO			177.5		
CH_3_CO				2.12, s	20.9
CH_3_CO					171.4

^a^ Measured at 400 MHz in CDCl_3_:CD_3_OD (10:1); ^b^ measured at 400 MHz in CDCl_3_; ^c^ measured at 150 MHz in CDCl_3_.

### 3.4. Viability Assay (FMCA Assay [25])

AGS cells were seeded in a 96-well culture plate (6 × 10^3^ cells per well) in 200 μL of Roswell Park Memorial Institute (RPMI) medium containing 10% fetal bovine serum (FBS). STF/293 cells (3 × 10^4^) were split into 96-well plates and incubated for 24 h. They were then incubated at 37 °C in a 5% CO_2_ incubator for 24 h. Test samples at different doses with or without TRAIL (100 ng/mL) were added to each well and incubated for 24 h. They were then treated with fluorescein diacetate (Wako, Osaka, Japan) in PBS buffer (10 μg/mL), and fluorescence was detected after a 1-h incubation. Assays were performed in triplicate at least. The sample was prepared as outlined above. Data are shown as the mean ± SD. The significance of differences between the data sets was determined with Tukey’s test (** *p* < 0.01 was regarded as significant). IC_50_ values were calculated by probit analysis.

### 3.5. Luciferase Assay

SuperTOP-Flash assay: Stable reporter cells, STF/293 cells (3 × 10^4^), were split into 96-well plates and grown in DMEM with 10% FBS for 24 h. Cells were treated with compounds and LiCl (final concentration: 15 mM). After the 24 h incubation, the cells were lysed with CCLR (20 μL/well cell culture lysis reagent; 20 μL/well, Promega, Madison, WI, USA), and luciferase activity was measured with a Luciferase 1000 Assay System (Promega, Madison, WI, USA).

SuperFOP-Flash assay: Transient transfection was performed using Lipofectamine 2000 (Invitrogen, Carlsbad, CA, USA). 293T cells (1 × 10^5^) were split into 24-well plates and grown in DMEM with 10% FBS for 24 h. After 24 h, cells were transfected with 1 μg of the luciferase reporter construct (SuperFOP-Flash) and 0.05 μg of pRL-CMV (Promega, USA) for normalization. Three hours after the transfection, compounds were added to the medium containing FBS. 293 T cells were treated with compounds in an FBS-containing medium combined with 15 mM of LiCl. Cells incubated for 24 h were lysed in passive lysis buffer (Promega , Madison, WI, USA, 50 μL/well), and luciferase activity was measured with a Dual-Glo Luciferase Assay System (Promega, Madison, WI, USA).

Luciferase chemiluminescence was measured using a Luminoskan Ascent (Thermo, Waltham, MA, USA). The assay sample was stored as a 10-mM solution in DMSO, then diluted to the indicated concentrations with medium. The final concentration of DMSO was less than 0.1% (v/v). Assays were performed in triplicate at least. Data are shown as the mean ± SD. The significance of differences was determined with Tukey’s test (** *p* < 0.01 *vs.* control). IC_50_ values were calculated by probit analysis.

## 4. Conclusions

In our research into the bioactive natural products in actinomycetes, we demonstrated the abilities of chromomycins A_2_ (**1**) and A_3_ (**2**) to overcome TRAIL resistance and inhibit TCF/β-catenin transcription. Chromomycin A_3_ has been shown to exhibit antitumor activity [9] and inhibit DNA synthesis by inhibiting DNA gyrase [26]. However, to the best of our knowledge, the effects of chromomycins on TRAIL resistance and Wnt signaling have not been reported. Therefore, these compounds may be potential drug leads for TRAIL-resistant and Wnt signal-related diseases and will be useful chemical tools for use in investigating TRAIL and the Wnt signaling pathway.
